# The development of animal welfare science in China: An explorative analysis

**DOI:** 10.1017/awf.2023.93

**Published:** 2023-10-27

**Authors:** Xin Guo, Franck LB Meijboom

**Affiliations:** 1 Science Technology and Pharmaceutical History Research Center, China Pharmaceutical University, Nanjing, China; 2 Faculty of Veterinary Medicine, Department of Population Health Sciences, Sustainable Animal Stewardship, Utrecht University, Yalelaan 2, NL-3584 CM Utrecht, The Netherlands; 3 Ethics Institute, Faculty of Humanities, Utrecht University, The Netherlands

**Keywords:** animal welfare, China, ethics, laboratory animals, livestock, public concerns

## Abstract

This paper presents results of a search and analysis of research projects on animal welfare registered in the China National Knowledge Infrastructure (CNKI) database in the period 1996–2019, with the aim of gaining a better understanding of developments in animal welfare science in China. The title-abstract search of publications in this database resulted in over 260 articles that could be linked to 200 research projects with an animal welfare component. These projects were analysed for: (a) involved academic disciplines; (b) studied animal species; (c) contexts of animal use; (d) concepts of animal welfare; and (e) attention to ethical dimensions of animal welfare. The analysis shows an increased attention to animal welfare science, with a particular focus on farm and laboratory animals. We observed an increase in the number of studies and of animal species studied. The majority of research projects start in or include a view of animal welfare that is close to Fraser’s ‘biological function’ view. We conclude that the increased attention to animal welfare in science reflects recent developments in China in terms of public concern about animal use, academic debate about the importance of animal welfare, and animal-related political and economic developments linked to China’s ambitions to be a global player in science and food production. For the further development of animal welfare science in China stable funding and more interdisciplinary collaboration are necessary to study and publish on fundamental aspects of animal welfare, on issues not directly related to applied problems, and on the ethical dimensions of animal welfare.

## Introduction

Animal welfare has developed into a key concept in the academic and public discussions on human-animal interactions, such as the use of animals for research, food or as companions, and is being recognised more and more at a global level (Bayvel *et al.* 2005; Fraser 2008; Food and Agriculture Organisation [FAO] 2014, 2018; Ryan *et al.* 2019). Nonetheless, the pace and scope of this development clearly differs between countries and regions. Whereas in the UK, the importance of a scientific basis to address animal welfare issues was recognised in 1926 by Charles Hume (Haynes 2008), it has taken until relatively recently in other parts of the world. Also, there are clear differences at a legal and policy level. In Europe, animal welfare has been embedded in national and European legislation since the mid-1970s (European Union [EU] 2014), while there are other countries in which the attention to animal welfare has not (yet) been included in legislation. This cannot simply be explained as simple frames that refer to national income or Western culture. Many middle-income countries, such as Costa Rica (since 1994), the Philippines (since 1998) and Tanzania (since 2008) have legislation on animal welfare.

Within this global trend China appears to be an interesting case study. China has not been a forerunner in its attention to animal welfare (Sinclair *et al.* 2020). Academic attention only started to be paid to animal welfare in the mid-1990s in mainland China (Li 2009). Even if one looks into Chinese tradition, the term ‘animal welfare’ cannot easily be recognised (Lu *et al.* 2013). To give an example, the concept of ‘husheng’ (护生) which translates as ‘protecting life’ (Poon 2019), can refer to animal welfare and animal protection. For example, it served as the basis for imperatives as regards to non-cruelty to animals during the Northern and Southern Dynasties (420–589) and was further developed in the Tang Dynasty (618–907). The concept is influenced by the Buddhist notion of benevolence to maintain the flourishing of all living things without interfering with the process of birth or life in nature. In practice, this means that ancient Chinese thinkers believed that people should not hunt animals or cut plants in spring and summer in order to allow plants and animals to grow and flourish. Also, in the 1930s, this concept of ‘husheng’ was used in an animal protection movement in China that was based on Buddhist activism (Schumann 2021). Thus, the concept of ‘husheng’ resembles important parts of what is meant by the Western concept of animal welfare but is also differs fundamentally in that it does not include the notion that animals are considered to be of value for their own sake, and has a wider scope that includes environmental issues.

Despite this conceptual issue, animal welfare and related concepts recently received more attention in the media and academic discussions in China (Qiu 2002; Qui 2004; Meng *et al.* 2012), and some empirical studies (You *et al.* 2014) also suggest a recent upsurge in attention towards animal welfare in the country.

This article aims to gain a better understanding of whether the increased awareness towards animal welfare is reflected in the development of animal welfare science in China. Based on a systematic search of projects and articles published and indexed in a central national database, we present data on the number and topics of Chinese research projects focusing on animal welfare. We then analyse and discuss the trends and the animal welfare concepts that appear to underlie these projects. Finally, we focus on the societal and ethical dimensions of animal welfare and discuss how this component can be better integrated into animal welfare research in China.

## Materials and methods

We conducted a desk review of nationally funded research projects on animal welfare in China, using the literature database of the China National Knowledge Infrastructure (https://oversea.cnki.net/index/) (CNKI; 中国知网). This is the largest open research database in China. It is managed and supported by the national government and provides full-text articles from over 2,000 Chinese journals, including doctoral dissertations, masters theses, conference proceedings, patents and newspapers.

To trace research projects on animal welfare, the decision was taken to begin with a search of published papers. We chose this approach because a title-abstract search of research projects would have potentially missed relevant projects since the title and abstract of research projects are relatively abstract. Papers as output of projects tend to be more specific, functioning as an entry-point to trace research projects that include an animal welfare research component. The CNKI database enables tracing of the research project(s) associated with these papers. Where a paper could be linked to a research project listed in the database, the project was included for further analysis.

As specific keywords for the title-abstract search, we used Chinese terms that refer to ‘animal welfare’ (动物福利) and ‘animal health’ (动物健康) with broader terms including ‘health’(健康), ‘healthcare’ (健康), ‘welfare’ (福利) and ‘ethics’(伦理) searched in combination with the search term ‘animal’ (动物). We deliberately included broader terms such as health as a search term because the concept of animal welfare is not easily translated into one Chinese concept. For instance, *fuzhi* (福祉) comes most close to the English term ‘well-being’. However, this is often used only with reference to human well-being. Therefore, a search with only *fuzhi* (福祉) in combination with animals as search terms would run the risk of missing relevant studies on animal welfare.

The first author (GX) searched the CNKI database for titles, abstracts and keywords of papers published between January 1980 and December 2020. The search was performed in 2019 and updated in 2021. This resulted in 266 articles containing one or more of the search terms indicating animal welfare research. These articles were linked to 200 research projects that included an animal welfare component, such as attention to animal behaviour, housing, or discomfort. These research projects were further analysed and reviewed for: (a) academic disciplines involved; (b) animal species studied; (c) contexts of animal use; (d) concepts of animal welfare; and (e) attention to the societal and ethical dimensions of animal welfare. The first three dimensions are listed in the database for each project. The fourth dimension is not predefined in the database. However, based on the analysis of the project titles and abstracts and the linked papers, it is possible to draw a first map based on the animal welfare orientations introduced by Fraser *et al.* (1997). This allows a distinction to be made between one view that sees biological functioning as the central element of welfare and a second that emphasises the affective states of animals in terms of pain, suffering and other feelings and emotions (both negative and positive). A third view is that animal welfare is about the ability of animals to live in the most natural circumstances possible, where they can express their normal behaviour. To be classified under the ‘biological functioning’ perspective, a project had to refer to welfare in terms of ‘health’, ‘growth’ and ‘productivity’. To be classified under the ‘feeling’ perspective, a project had to describe animal welfare in terms of ‘fear’, ‘pain’, ‘stress’ and ‘pleasure’. Finally, terms such as ‘natural’, ‘free-roaming’ and ‘wild’ led us to classify a project as one that included a definition of animal welfare in terms of natural living.

## Presentation of the data

### Number of projects

**Figure 1.**
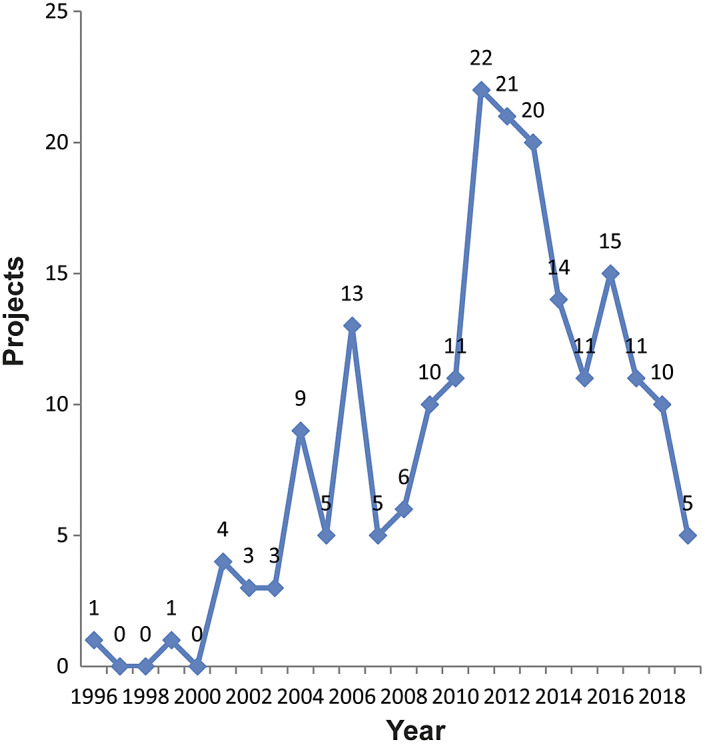
Number of animal welfare projects funded by the Chinese government between 1996 and 2019.

We identified 200 funded research projects that included an animal welfare component. The data show an increase in the number of published research projects on animal health and welfare in China since 2000 (Figure 1). This suggests that there was limited formal funding for animal welfare in research published before 2000, although a few individual projects were funded. From 2000, there is a steady increase in the number of funded research projects in China until 2011. From 2013, the data indicate a decline in newly funded animal welfare projects here. However, it is too early to claim whether this represents a true decline or whether we are approaching a steady state. To understand Figure 1, it is important to bear in mind that most projects are funded for a period of five years, which means that the high number of funded projects in 2011–2013 will continue until 2016–2018. Furthermore, due to our search method, which starts with a title-abstract search of papers, the data on projects funded in 2018 and 2019 are less accurate, as it takes time for the results to be published and the projects granted in these years of our search may have been affected by the COVID-19 outbreak.

### Disciplinary background of the research projects

Animal welfare is an academic field that combines contributions from several disciplines, including biology, ethology, physiology, veterinary science and ethics. Table 1 shows the disciplines involved in published research projects and how they are represented. For this characteristic, we used the disciplinary categories listed in the research application, such as biology, biochemistry, animal science, veterinary medicine, food science and technology, agricultural economics and management science. In addition, if the project summary or publication abstract explicitly mentioned the use of other disciplines, we included this in our analysis.

**Table 1.** Main disciplinary focus of animal welfare research projects funded by the Chinese government in the period 1996–2019

Disciplines	Projects (total n = 200)	Percentage
Animal (Farming) Science	37	18.5
Animal Physiology	19	9.5
Animal Cognition	4	2.0
Animal Ethology	10	5.0
Animal Ecology	11	5.5
Food Science and Technology	13	6.5
Veterinary Science	23	11.5
Environmental Science	12	6.0
Toxicology and Medicine	10	5.0
ICT	7	3.5
Animal Housing Systems	4	2.0
Economic and policy studies	23	11.5
Philosophy	10	5.0
Law	17	8.5

The data show that, in addition to animal ethology (5%) as an essential component of animal welfare research, agricultural and animal science (18.5%), veterinary science (11.5%) and economic and policy studies (11.5%) play an important role in Chinese animal welfare research. A wide range of other disciplines play a less prominent role, such as philosophy, animal psychology and ICT science.

### Contexts of animal use and animal species

**Figure 2.**
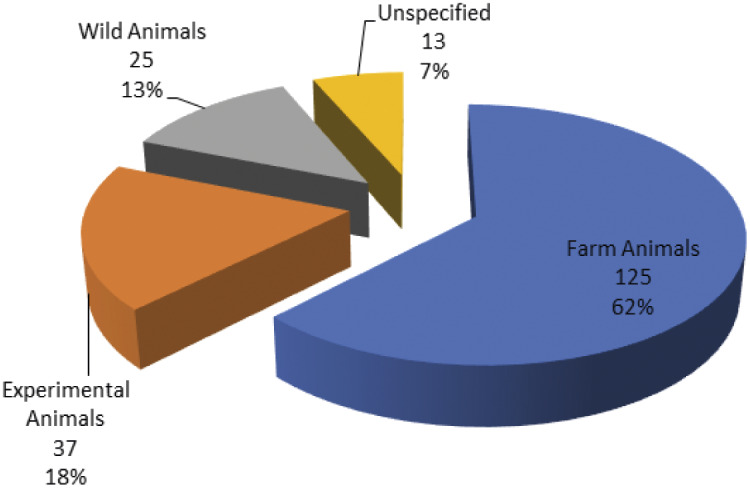
Percentage of animal welfare research projects in China relative to animal use (1996–2019).

**Figure 3.**
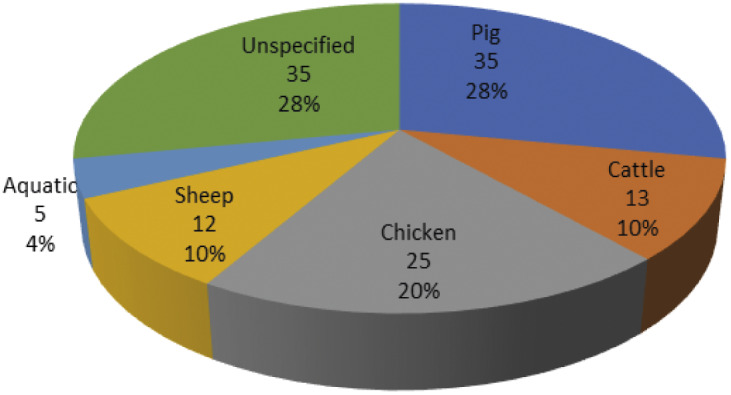
Percentage of animal species studied in animal welfare projects in livestock farming in China in the period 1996–2019.

The data point to trends in the contexts of animal use and animal species that are covered by the research projects. The majority of the projects focus on animal welfare in the contexts of farming, laboratory animal testing and wildlife (Figure 2). The projects on livestock farming, mainly study the health and welfare of pigs, poultry, cattle and sheep (Figure 3). In the context of laboratory animal sciences, the projects focus primarily on rodents, especially mice. Only fish and sheep are studied in all three contexts: farming, laboratory animal testing and wildlife management. The current data indicate the absence of animal welfare research in the contexts of entertainment and education (e.g. zoos) and companionship in China. As a consequence, animals such as dogs or large wild animals are rarely subject of the analysed projects. The data reflect that published animal welfare research in China focus mainly on those species relevant for food production, public health and science.

### Views on animal welfare

**Figure 4.**
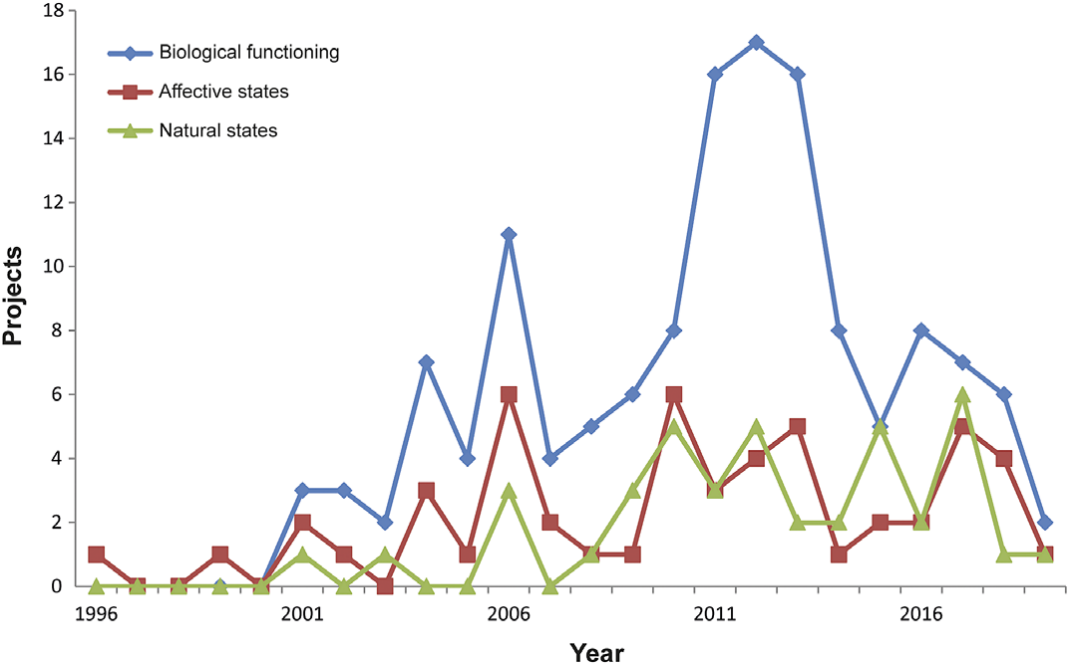
Chinese animal welfare research projects classified in one or more of Fraser’s quality of animal life concerns by year (Fraser *et al.* 1997; Fraser 2003a).

In conjunction with the above-mentioned point that the concept of animal welfare is not easily translated into a single Chinese concept, the data also suggest diversity in the way animal welfare is defined and assessed in the projects. We explored the views on animal welfare underlying the research projects. Most projects do not provide explicit definitions, either in the published papers or in the research description. Nonetheless, the analysed material provides an indication of how animal welfare is interpreted and made operational. Rather than searching for specific definitions, we used Fraser’s framework of animal welfare concepts. He defines three general views regarding the welfare of animals (Fraser *et al.* 1997; Fraser 2003a,b). Although readily distinguishable, they should be understood as overlapping animal welfare concerns (Fraser *et al.* 1997). In line with previous research (Spooner *et al.* 2012, 2014), we used this framework to analyse how animal welfare was perceived in the research projects. The analysis showed that a number of projects referred to more than one of the three views, consistent with the idea that these are not mutually exclusive views of welfare. Therefore, the total number of projects linked to one of the welfare concerns in Figure 4 is higher than the total number of projects that we studied.

Analyses indicate that most projects include a view of animal welfare that takes the biological functioning of the animal as the central element of welfare. This is reflected in papers describing welfare in terms of ‘health’, ‘growth’ and ‘productivity’. The other two views are clearly less prevalent. Nonetheless, the figure shows a steady number of projects that include the natural state and affective state views of animal welfare.

### Societal dimensions of animal welfare

Since animal welfare is more than a biological concept and includes ethical and societal dimensions (Haynes 2008; Ohl & Van der Staay 2012), we analysed whether and to what extent projects addressed this aspect of animal welfare. From those included, 50 refer to the societal dimensions of animal welfare, i.e. the research proposal or the related output explicitly mention the legal, philosophical, economic, policy or sociological dimensions. From this selection of projects only 30 and 33 papers include funding to study the societal dimensions. Further scrutiny reveals projects that explore how humans interact with nature from an ecological perspective (n = 10), those probing the need for new legislation related to animal welfare (n = 9) or that focus on international animal welfare regulations (n = 3), deal with the ethics of animal testing (n = 3), study the relationship between food safety and animal farming (n = 5), analyse consumers’ willingness to pay for increased welfare (n = 2) and explore the societal context of developments in livestock farming (n = 3).

## Discussion

Our strategy of starting the search in published articles and linking these to funded research projects has undoubted limitations. For instance, the first Chinese article concerning animal welfare had already appeared in 1984 yet it was not included in our analysis because a national project on this topic was not funded until 1996. A similar problem appears at the end of the search period. Animal welfare projects were funded in 2020 and 2021, but most of the results of these projects were yet to have been published when our search was conducted in 2021. Therefore, it is not yet possible to attain an accurate picture for these years and they are excluded from our study. Despite such limitations, our search encompasses a period of more than 20 years (1996–2019), which is sufficient to explore and analyse trends in published animal welfare science. Another limitation is that the CNKI data do not cover all animal welfare research in China and we focused on published papers. However, the database used is one of the most relevant and accessible sources in China. Focusing on published work seems the only feasible way of systematically tracking relevant projects. The CNKI is a publication- rather than a project-oriented database. We consider the risk of missing projects to be relatively low, as we have already identified projects when there was a co-publication (e.g. with another project). Furthermore, we do not claim the results to be representative of all welfare research in China, but rather that they illustrate trends and developments in the field.

Based on the analysis of the data we formulate five points for discussion: (a) the context in which the increased number of animal welfare research project is embedded; (b) the change in research focus; (c) the central position of farm animals; (d) the ‘biological function’ view as the main perspective on animal welfare; and (e) the limited attention to the societal and ethical dimensions of animal welfare.

### Increased number of research projects in context

The data show an increase in the number of animal welfare research projects. We have not found one single cause that would fully explain this development. Nevertheless, it reflects recent developments at three levels: public concern; scientific debate; and political and economic interests.

From a public perspective, attention to animal welfare is a relatively new issue in China. Until 2000, the issue did not receive much attention (You *et al.* 2014). This has changed; animals, and in particular their welfare, have become part of the public debate (Lu *et al.* 2013; Li 2006; Li 2009; Su & Martens 2017; Carnovale *et al.* 2021). Social media platforms see animal abuse and cruelty explicitly condemned, and the vulnerable position of animals and their welfare addressed via a range of public activities. These activities contribute to a broader critical debate regarding whether (increased) human welfare should come at the expense of reduced animal welfare (Loeffler *et al.* 2009; Meijboom & Li 2015). A shift in public attention to animal welfare is also reflected in a survey of citizens’ perceptions of farm animal welfare in China (You *et al.* 2014). The survey shows that 44% of respondents harbour concerns about current factory farming. From this group, 23.8% are of the opinion that this form of livestock farming seriously curtails the freedom of pigs and poultry and 20.2% regard current production practices as cruel towards pigs and poultry. Of the respondents, 65.8% completely or partly agree on establishing mandatory laws for animal welfare that would compel producers to provide better living conditions for farm animals (You *et al.* 2014).

Also, in academia, animal welfare was subject to greater attention. In parallel to a rise in the number of published funded research projects in the early 2000s, animal welfare has also been part of an academic debate that has focused on the relevance of and need for research, legislation and policy-making for animal welfare. In this debate, two main perspectives can be recognised. On the one hand, there is a plea to further reflect on animal welfare in research and legislation (Qiu 2004). On the other, some argue that welfare concepts focusing on individual animals mainly ascribe to Western concepts that are not compatible with the Chinese tradition of caring for animals and do not fit the Chinese situation (Zhao 2004).

Finally, the increased attention to animal welfare is linked to economic and political developments. To start at the economic level, as a global player in science and food production, China is required to comply with international standards that include views on the use of animals. For example, when China joined the World Trade Organisation (WTO) in 2001, there were health and food safety requirements (Littlefair 2012). Now that it is the largest livestock-producing nation in the world (FAO 2021), expectations also include standards for animal health, including aspects of zoonoses and animal welfare (Sinclair *et al.* 2022). Attention to animal welfare can also be seen at governmental level. For instance, as part of the 9^th^ Five-Year plan (Ministry of Science and Technology [MOST] 1997), the Chinese government issued a programme to highlight welfare of laboratory animals and introduced the 3Rs principles (Replacement, Reduction, Refinement). Moving forward, the guidelines were updated in 2001 (Kong & Qin 2010) and replaced with a novel set issued in 2018. These present the need for ethical assessment and animal welfare in the context of animal experimentation. In this document, animal welfare is defined in terms of the Five Freedoms (Brambell 1965). Additionally, animal welfare research forms part of a more general development. In 2012, the 16th National Congress of China introduced the vision of an “ecological civilization” (Shengtai Wenming, 生态文明) as a guiding principle for sustainable development (Gu *et al.* 2020; Xiao & Zhao 2017). This vision reflects concerns about the relationship between economic and social development and ecological risks. The official narrative relies upon a series of metaphors, starting with the agricultural civilisation, characterised by the colour yellow, the colour of the soil in Chinese tradition, and based on the notion of harmony between humans and nature (Pan 2016; p 35). Next, comes the industrial civilisation, which is associated with the colour black, representing coal and steel. The current vision for an ecological civilisation is characterised by green and aims to nurture a relationship between humans and nature capable of responding to actual economic and climate challenges. This includes the development of animal husbandry in a more sustainable way, where humans, animals and nature live in harmony.

The above-mentioned developments in public concern, scientific debate and political and economic interests should not be seen as direct causes of the increase in the number of animal welfare research projects, but they do illustrate the Chinese context into which the increase fits and can be seen as a logical response.

### Changes in research focus

The data illustrate that animal welfare science in China started with a focus on ethology, e.g. ethological research on animal welfare problems in sows in 1996. This starting point can be explained because ethology is one of the basic elements of animal welfare and therefore serves as an essential starting point for building a research portfolio in animal welfare science. It can also be explained by the personal background of the principal investigators of the projects that started prior to 2000. For example, one of the pioneers, Bao Jun, completed his PhD at University College Cork and was trained in a tradition of animal welfare science that included attention to ethology. As a result, early projects were concerned with fundamental issues, such as a 2001 project entitled ‘Systematic study on the communication behaviour of Sichuan golden monkeys’. However, from the outset, basic animal welfare research has been linked to more applied issues in farm and laboratory animal research. For example, in 1999, a project was instigated looking into mechanisms that underlie stereotypical behaviour in sows. With the increase in research projects in 2006 and 2011, we saw a further move towards more applied issues related to farming or animal testing. This also had a direct impact on the disciplinary focus of the projects (see Table 1). This change can be understood in the context of China’s rapid economic development into an industrialised country and a global player in livestock production. Science and technology are seen as important components of this process. This also applies to animal welfare science. Although the importance of animal welfare science as such is not questioned, the research focus seems to be driven by the potential contribution to the development of a modern livestock system and increased production rates. This is not only driven by economic considerations, but also by the increase in meat production and consumption in China (Sheng & Song 2019). For the last period analysed in this study (2017–2019), a number of papers indicate a further shift in research focus. As a result of the global outbreak of COVID-19 and the widely held view that it originated in wet markets, animal welfare in relation to food consumption and wildlife conservation was investigated (Xiao *et al.* 2021).

### Main focus on farm animals

The data show that the majority of the funded research projects were concerned with animal welfare questions related to livestock farming. This is also consistent with other published research on animal welfare in China (Sinclair *et al.* 2020; Yin & Zhu 2020). This can be partly explained by the points raised in the previous section. However, two additional points are important in understanding this focus on farm animals.

First, there is a lack of animal welfare regulation for livestock production in China. While the Ministry of Science and Technology introduced the concept of animal welfare and the 3Rs to guide the practice of animal experimentation as early as 1997 (MOST 1997), followed by the Guideline on the Humane Treatment of Laboratory Animals in 2006 and 2018 (Standardisation Administration of China 2018), there is no parallel development in the context of agriculture.

Second, China has roughly two completely different types of animal farming that exist in parallel. On the one hand, China is one of the largest livestock-producing nation in the world. As far back as 2012, Nielsen and Zhao estimated that “in the last decade China has secured its position in pig production, having more than 50% of all pigs in the world” (Nielsen & Zhao 2012) and that is unlikely to change, even taking into account the problems with African Swine Fever (Yu *et al.* 2019). As a result, many pigs are kept in intensive and industrial systems. Therefore, all kinds of well-known animal welfare challenges that are associated with breeding, housing and slaughter of high numbers of animals are also applicable to the Chinese situation, such as the inability to express species-specific behaviour, aggression and forms of maladaptation. Research has therefore been funded to address and mitigate these problems. On the other hand, although the official trend is towards intensification, pigs in China are still kept on small farms in less-developed rural areas (Zhou *et al.* 2014; Sinclair *et al.* 2020). Although animals on these farms may have more opportunity to express species-specific behaviours and live at a reduced densities, they are also at risk of welfare problems. On these small farms, health status and feed quality are often lower, or at least less standardised.

This juxtaposition of industrial and more traditional animal husbandry gives rise to diverse and complex animal welfare issues. This explains the strong focus on farm animals and also highlights a more recent development. In the last years of our search period (2017–2019), we found that the ‘National Key Research and Development Program of China’ has funded six research projects on animal welfare in pig and poultry farming, addressing welfare as part of future farming. One project explores animal welfare problems as a reason to search for plant-based meat alternatives (Yu *et al.* 2022).

### Main focus on ‘biological function’

The data as presented in Figure 4 show that most research projects start with or include a view of animal welfare that is close to Fraser’s ‘biological function’ view (Fraser 2003b). This can be explained by the above-mentioned focus on farm animals. In this context, the most urgent animal welfare issues are still perceived as problems related to the biological function of animals, such as health, growth and reproduction. Secondly, the study of affective states such as positive emotions is extremely challenging. This is not an easy start for a young field of research, as it was for China in the early 2000s, and still remains a matter of debate for research groups with more experience in animal welfare research on how best to study these dimensions of positive animal welfare (Lawrence *et al.* 2019). Finally, the focus on the health and functional dimensions of animal welfare may also reflect a more instrumental view of the moral position of animals. Although there are clear trends in China showing that the societal position of animals is changing (Li 2006; Barber & Hathaway 2022), there are still clear differences in how animals are valued in politics and society. From the perspective of the state, animals do not have a clear moral or legal position. This is not just a result of current politics but has a longer philosophical tradition. For instance, in Confucianism, the position of animals is diffuse, and some argue that Confucians “at most support limited animal welfare only, but they definitely deny animal rights (Chan 2016; p 473). In practice, attention to animal welfare is more easily accommodated by economic considerations, which are more in line with the ‘biological function’ perspective on animal welfare.

### Limited attention to the societal and ethical dimensions of animal welfare

The attention to societal and ethical dimensions is limited. Only 30 projects (15%) show a link with disciplines other than those related to (veterinary) medicine and biology. Within this group, the number of projects that deal directly with societal and ethical dimensions of animal welfare is even smaller (n = 6). More academic attention is given to scientific knowledge and techniques rather than to the social and normative dimension of animal welfare. On the one hand, one could argue that this should come as no surprise. The scientific study of animal welfare is a prerequisite for any further analysis of the societal or ethical dimensions. It seems logical therefore to start with a strong focus on the veterinary and biological dimensions. However, as has been argued previously, animal welfare is more than a biological concept (Ohl & van der Staay 2012). Furthermore, there is no clear division of labour, with scientists working only on the biological dimensions of welfare, while social scientists or ethicists work on the societal dimensions. Animal welfare research requires an integrated and interdisciplinary approach. As Fraser puts it, “to address ethical concerns about the treatment of animals, scientists needed ethical reflection to complement their empirical information; and ethicists needed to ground their arguments in sound knowledge of animals and animal use practices” (Fraser 1999; p 173). However, such interdisciplinary approaches are still limited in China. In addition, ethical reflection is needed when there are moral problems. However, government policies, such as state goals based on the principles of ecological civilisation, already guide actions and suggest that there is less need to reflect on the societal or ethical dimensions of animal welfare. Finally, it is important to note that the traditional meaning of the word ‘hehui’ (社会), which stands for society or social, refers to humans and the interaction between them. From this perspective, animals are included in societal interaction mainly as a resource or in production roles, rather than as a reason to reflect upon their individual position, as we mentioned in the previous section. Nevertheless, greater attention to the societal dimensions of animal welfare could improve research quality, outcomes and implementation, as this type of research helps to elucidate the beliefs and attitudes of relevant stakeholders, including the general public, even in the case where government targets have been set. Furthermore, it is especially important within the Chinese context, since it allows a discussion on how Western views on animal welfare relate to (traditional) Chinese perspectives on the human-animal relationship and animal welfare (Meijboom & Li 2015).

## Animal welfare implications and conclusion

With this paper we aimed to gain a better understanding of the development of animal welfare science in China. Based on the systematic search and analysis of a selected set of relevant projects and articles, we conclude that there has been an increase in the attention to publishing animal welfare science in the period 1996–2019. Not only was there an increase in the number of studies, but also in the diversity of those species studied. As knowledge is an essential first step in improving animal welfare, this is an important development. To further improve the quantity and quality of animal welfare science and its practical implications for animal care, it is important to understand the background to this increase in research attention to animal welfare. Our analysis of research projects shows it to be a reflection of recent developments in public concern about animal welfare and animal use, an academic debate on animal welfare, and animal-related political and economic developments linked to China’s ambitions to become a global player in science and food production. This exploratory study shows most research to be problem- rather than curiosity-driven. The focus falls predominantly on farm animals and starts with a functional view of animal welfare. Given the challenges China faces in terms of food security, food safety and sustainability, this may be understandable and effective start. However, it would be good for the future development of animal welfare science in China if there were stable funding and more interdisciplinary collaboration for research and publication on fundamental aspects of animal welfare, on issues not directly related to applied problems, and on the societal and ethical dimensions. This does not diminish the importance of the research undertaken in the previous 25 years but can contribute to the further development of animal welfare science as a stable and mature field of research.
